# Effectiveness of Using Compression Garments in Winter Racing Sports: A Narrative Review

**DOI:** 10.3389/fphys.2020.00970

**Published:** 2020-08-04

**Authors:** Chenhao Yang, Yongxin Xu, Yang Yang, Songlin Xiao, Weijie Fu

**Affiliations:** ^1^School of Kinesiology, Shanghai University of Sport, Shanghai, China; ^2^Key Laboratory of Exercise and Health Sciences of Ministry of Education, Shanghai University of Sport, Shanghai, China

**Keywords:** speed skating, alpine skiing, cross country skiing, air drag, muscle function, strength, fatigue, metabolism

## Abstract

Nowadays, compression garments (CGs) are widely used in winter racing sports, such as speed skating, short-track speed skating, alpine skiing, and cross-country skiing. However, the effect of wearing CGs on athletic performance in these specific sports is still not fully examined. Thus, the aim of this narrative review is to summarize the research and application of CGs in winter racing sports and to discuss how the CGs help athletes improve their performance in an integrative manner (i.e., physiology, aerodynamics, and biomechanics). A total of 18 experimental studies dedicated to CGs in winter racing sports were identified from the peer-review scientific literature. The main findings are as follows. (1) Currently, CG studies have mainly focused on drag reduction, metabolism, muscle function, strength performance, and fatigue recovery. (2) The results of most studies conducted in wind tunnels showed that, for cylindrical structures similar to the human body, clothing with rough surfaces can reduce air drag. Notably, the effect of CGs on drag reduction in real competition has not been fully explored in the literature. (3) Compression can reduce muscle vibrations at high impact and help athletes control the center of pressure movement, a function that is important for alpine skiing. Future studies are needed to improve current understanding of the effects of compression clothing microstructure on drag reduction and their stretching in different parts of the body. Furthermore, the design of experimental protocol must be consistent with those during the competition, thus providing a full discussion on energy metabolism, fatigue, and recovery affected by CGs.

## Introduction

In modern sports events, sport equipment is an essential factor for athletes to overcome their limits and achieve breakthroughs. Nowadays, high-tech compression garments (CGs) (e.g., compression clothes, shorts, and socks) are becoming increasingly popular among sports elites and enthusiasts. Meanwhile, the physiological benefits of CGs in enhancing athletic performance have been widely recognized, i.e., improvement of fatigue recovery (Brown et al., 2017), promotion of blood flow (Privett et al., 2010), regulation of blood lactate and creatine kinase levels (Kim et al., 2017), and the enhancement of muscle functions (e.g., reducing muscle vibrations and activation) (Broatch et al., 2019). Aside from the aforementioned advantages, CGs have also been commonly used and have shown potential benefits in winter racing sports (Sperlich et al., 2013; Chowdhury et al., 2015; Moon et al., 2016), including speed skating, short-track speed skating, alpine skiing, cross-country skiing, bobsleigh, luge, and skeleton. These broader applications can be attributed to the integrated aerodynamics and mechanical properties of the latest CGs.

In particular, the aerodynamic application of CGs is critical for winter racing sports. For example, speed skating (including short-track) generally reaches an average speed of 35–40 km/h (Brownlie and Kyle, 2012); meanwhile the average speed of alpine skiing, bobsleigh, and luge can exceed 100 km/h (McCradden and Cusimano, 2018). Therefore, the design principles of individualized CGs for each discipline have been set according to the peculiarity of aerodynamics (Supej and Holmberg, 2019). Furthermore, small differences in aerodynamic drag can exert a major impact on finishing time (Supej and Holmberg, 2019). Similar to dimpled golf balls, these racing CGs use common textured fabrics, and the structure could trip the wake turbulence flow to reduce drag. Chowdhury et al. (2015) reported a rough fiber structure of skating suits, which showed a significant positive effect on the air flow characteristics over the surface. In addition, bobsleigh, skeleton, and luge athletes use either their body posture or rudder to control the direction of their vehicles’ slide on the ice track (Mosey, 2014; Colyer et al., 2018). Therefore, the primary task in those three events is to reduce the drag of the track and air by changing body posture and using proper clothing and equipment, thus increasing the transmission efficiency of the system (Peyre-Tartaruga and Coertjens, 2018). This can be achieved, for example, by covering the skin using CGs to reduce the drag area of the body (Spring et al., 1988). Collectively, the fabric surface design/materials of CGs can be regarded as an aerodynamically beneficial approach, which can help optimize the aerodynamic drag of racing sports to the greatest extent (Bardal and Reid, 2012).

On the one hand, in terms of mechanical properties, CGs can stabilize/support the underlying tissue by reducing the vibration of the soft tissue compartments (Sperlich et al., 2013). This can help mitigate exercise-induced discomfort and potentially reduce the energy expenditure, which largely depends on the pressure applied to the skin and musculature (MacRae et al., 2011). On the other hand, with respect to the energy production and muscle efficiency, CGs can provide multiple benefits, i.e., reducing muscle fatigue (Bringard et al., 2006), accelerating recovery of muscular power (Kraemer et al., 2010), and promoting the motor-unit activation coordination (Moon et al., 2016). Furthermore, CGs may optimize sports techniques; for example, the prediction of performance can be determined by the aforementioned parameters of energy production, efficiency, and technique with a power balance model (de Koning et al., 2005). Thus, these improved physiological and biomechanical characteristics brought on by the use of CGs are beneficial for enhancing athletes’ performance in winter racing events (Supej et al., 2011).

The aim of this narrative review is to examine the effectiveness of using CGs in winter racing sports. We will begin by reviewing the drag reduction performance of CGs. Then, we will assess the effect of CGs on physiology and biomechanics. This will be followed by a review of how CGs influence fatigue recovery. Finally, we will identify gaps in our knowledge and provide suggestions for future directions. The overall aim of this paper is to summarize the research and application of CGs in winter racing sports and to discuss how the CGs help athletes improve their performance in an integrative manner (e.g., aerodynamics, physiology, and biomechanics).

## Literature Search Methodology

While this review is narrative in nature, a systematic search was conducted via Web of Science, PubMed, and SPORT Discus (EBSCO) from inception to January 2020 in order to ensure that relevant studies were not overlooked. Articles were required to be peer-reviewed, in full text, and in English language. The keywords for the search were “speed skating,” “bobsleigh,” “luge,” “skeleton,” “alpine skiing,” “cross-country skiing,” “compression suits,” “competition suits,” “skinsuit,” “compression garment,” and “compression clothing.” Search terms were combined by Boolean logic (AND, OR). Research articles were included if they (1) analyzed CGs for winter racing events and (2) were about sports science and sport medicine. Articles were excluded if they (1) were review articles, business reports, and official announcements; (2) were about cloth design, textile technology, and social sciences; and (3) had duplicate and ambiguous literature. The reference lists of all the articles retrieved were also manually searched for any relevant articles that were not identified electronically.

**FIGURE 1:**
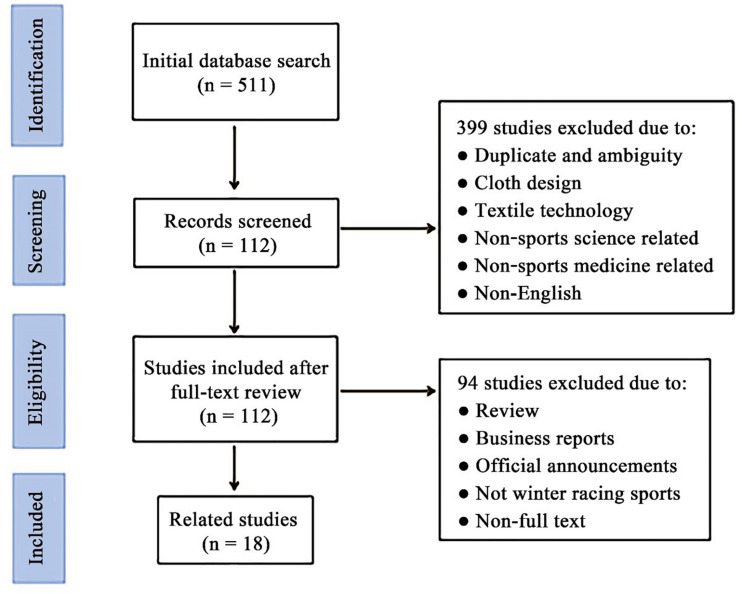
Flow diagram of the literature selection process and the number of articles (n) after each stage.

**FIGURE 2:**
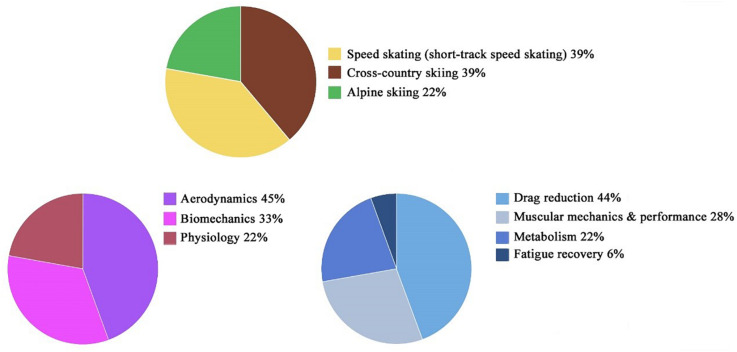
Classification and proportion of research on CGs for winter racing sports.

To date, there have been thousands of studies on pressure garments or CGs in various fields. Initial results were retrieved in more than 500 publications. The authors determined the exclusion criteria by reading titles and abstracts that fit the inclusion criteria, resulting in 112 articles. After reading the full texts of these articles, only 18 experimental studies were identified to be dedicated to CGs in winter racing sports (Figure 1). After simple classification and statistics, we found that these studies involved aerodynamics, biomechanics, and physiology. The studies were mostly focused on aerodynamic research on drag reduction (44%), metabolism (22%), muscle mechanics (28%), and fatigue recovery (6%) (Figure 2). In the current study, we did not systematically review these articles. However, we conducted a narrative review of the articles and combined their different aspects, such as integrative physiology and biomechanics.

## Findings

As shown in the searched results (Table 1), the effectiveness of using CGs in winter racing sports was from an integrative perspective, i.e., drag reduction, metabolism, muscle mechanics, and fatigue recovery. From the perspective of mechanical efficiency, two main aspects were proposed: the transmission/propulsive efficiency (drag reduction, joint stability, etc.) and the muscle efficiency (muscle fatigue, motor-unit activation coordination, etc.). Specifically, in the drag reduction functions, wind tunnel experiments were usually conducted according to the speed, posture, and anthropometry in various events. The majority of studies have shown that CGs with appropriate rough surfaces can reduce air drag (Brownlie et al., 2004; Oggiano and Sætran, 2010; Bardal and Reid, 2012). Meanwhile, CGs bring about benefits in physiology and biomechanics by applying compression to the skin and musculature, which can be achieved through the improvement of metabolism (Sperlich and Holmberg, 2011), the reduced muscle vibrations at high impact (Sperlich et al., 2013), and the improvement of muscle function (Moon et al., 2016). However, the number of studies on winter racing sports is not as many as those on general sports, and the fundamentals of the theory have yet to be fully explored.

**TABLE 1:** List of studies on the effectiveness of using CGs in winter racing sports.

**Author**	**Year**	**Population**	**Compression suit**	**Study design**	**Findings**
**Drag reduction (*n* = 8)**					
Brownlie et al., 2004	2004	A mannequin model based on scale photographs from 15 elite sprinters and 16 speed skaters	Fitting samples of 39 stretch fabrics	(1) *F*_D_ and air velocity were measured with wind tunnel tests. (2) *C*_D_ and Re were used to characterize the ability of the various fabrics to generate FT.	Compared with the bare leg, the leg model covered with the best-matched fabric reduced *F*_D_ and *C*_D_ by 20.2 and 40%, respectively.
Kuper and Sterken, 2008	2008	Full set of the speed skating results of the 2002 Olympic Winter Games	Five branded skating suits	The average skating speeds of the 500–10000 events were modeled with a pooled linear regression. The two-stage generalized least squares (instrumental variable) of regression model are the home advantage dummy variable, age, length, squared length, and weight of the athletes.	Once corrected for selection bias, none of the suits outperformed the others.
Sætran and Oggiano, 2008	2008	A mannequin was used to simulate the average posture of 1500 m speed skating athletes.	Six suits with fabrics of different roughness for lower legs, upper legs, trunk, arms head, and arms.	The speed of each discipline was simulated for women and men, and air drag was measured using the sensors placed at the joints.	(1) The final time difference of about 3 s was estimated in the suits with different surfaces. (2) Legs were the most sensitive area in fabric selection.
Oggiano and Sætran, 2010	2010	A mannequin was used to simulate the posture of cross-country skiing athletes.	Six suits with three kinds of roughness and two kinds of thickness.	Velocity varied from 15 m/s to 25 m/s with 1 m/s interval. Force balance in the base plate measured the air drag.	Ski suits with rough fabrics reduced air drag by 10%.
Oggiano et al., 2012	2012	A cylinder model simulated the limbs.	Three cross-country suits with rough fabrics were used, which were stretched at 9, 23, 43, and 63%.	The range of test velocity was 0–16 m/s. Force balance in the base plate measured the air drag.	A higher strain “smooths” out the fabric surface, moving the critical speed closer to the speed for a smooth one.
Brownlie and Kyle, 2012	2012	A mannequin of a competitive speed skater was used.	Six speed skating suits used in the 2002, 2006, and 2010 Winter Olympic Games.	(1) *F*_D_ measurements were performed with wind tunnel tests in the air velocity range of 12–15.6 m/s. (2) The difference in pre-Olympic to Olympic performance based on skating suits was compared.	(1) SWIFTSkin was observed to provide the greatest reduction in *F*_D_ by 13.3%. (2) Skaters with SWIFTSkin suit exceeded their previous personal best performance by 1.03%. (3) Skaters clad in other speed suits exhibited minor differences in performances.
Bardal and Reid, 2012	2012	The cylinder models with three different diameters were used to model the calf, thigh, and upper arm	With stretch of 25 and 42%, the fabrics with three different roughness levels were compared.	The fabric performance under stretching were tested in the wind tunnel at an air velocity equivalent to 17.5 m/s at typical race environmental conditions	The transition speed was affected when the absolute stretching of the fabrics is increased from 25 to 42%
Chowdhury et al., 2015	2015	A standard cylinder was used with a 110 mm diameter and 200 mm length	Four commercially made skinsuits from different manufacturers were compared.	Wind tunnel tests were conducted at a range of air velocities (20–120 km/h with an increment of 10 km/h). Electron microscopic analysis was used to observe the fabric properties of the skinsuits.	(1) Compared with the bare cylinder, all skinsuit fabrics can gain aerodynamic advantages. (2) Smooth surface for streamlined bodies can reduce the drag efficiently. (3) An increase in surface roughness can reduce the drag for the quasi-cylinder.
**Metabolism, muscle function, and mechanical performance (*n* = 9)**					
Sperlich and Holmberg, 2011	2011	Six elite cross-country skiers (age: 29 ± 6 years; height: 174 ± 10 cm; body mass: 67.9 ± 10.7 kg)	2009 and 2010 racing suits	(1) Tested at 12 km/h at 5° inclination; 11 km/h at 6° inclination; and 12 km/h at 8° inclination with roller skating for 6 min. (2) Tested oxygen uptake, minute ventilation, HR, skin, core temperature, etc.	(1) Oxygen uptake, minute ventilation, RER, HR, etc. were all lower with the 2010 skiing suit. (2) Average skin and core temperature were lower with the 2010 skiing suit.
Sandsund et al., 2012	2012	Nine male endurance athletes (age: 25 ± 3 years; height: 183 ± 7 cm; body mass: 78.6 ± 7.4 kg; body fat: 11.9 ± 2.3%; VO_2max_: 5.6 ± 0.4 ⋅ min^–1^)	With one-piece cross-country skiing suits	(1) Standard running tests were performed at six ambient temperatures (–14, –9, –4, 1, 10, and 20°C) with an air velocity of 5 m/s. (2) Skin and core temperatures, VO_2max_, TTE, running economy (HR and VO_2_), and running speed at LT were measured.	(1) Skin temperature decreased significantly with reduced ambient temperatures, and core temperature increased during all conditions. (2) Optimal endurance performance with cross-country skiing suit was found at –4 and 1°C.
Sperlich et al., 2013	2013	Twelve elite male alpine skiers (age: 26 ± 4 years; body mass: 80 ± 5 kg)	Three compression shorts in different pressures (0, 20, and 40 mmHg)	(1) Simulated actual alpine skiing posture, vibration and load for 3 min. (2) Tested EMG activity, cardiopulmonary data, hemoglobin, oxygenation of VL, muscle vibration, blood lactate concentration, RPE, joint angle, maximal isometric knee flexion, jumping height, and balance ability before, during, and after trials.	(1) Knee flexion and muscle vibration decreased with increasing pressure. (2) Percentage of muscle activities (pre–post): TA (20.2–28.9%), gastrocnemius medial (4.9–15.1%), RF (9.6–23.5%), and VM (13.1–13.7%). (3) Hemoglobin without compression was lower than 20 or 40 mmHg compression.
Born et al., 2014	2014	Ten German speed skaters from the national team (age: 23 ± 7 years; height: 173 ± 10 cm; body mass: 68.2 ± 13.9 kg; peak oxygen uptake: 58 ± 5.2 mL ⋅ kg^–1^ ⋅ min^–1^)	With or without compression shorts	(1) Simulated the 3000 m speed skating competition. (2) Tested the lateral femoral muscle oxygenation and blood volume with near-infrared (IR) spectroscopy. (3) Tested the oxygen uptake, ventilation, HR, speed, etc.	(1) Oxygenation in the vastus lateralis was asymmetrical. (2) Leg compression did not affect oxygenation asymmetry and other parameters.
Sperlich et al., 2014	2014	Ten elite long-distance cross-country skiers (age: 25 ± 4 years; height: 180 ± 4 cm; body mass: 74.6 ± 3.2 kg)	With or without long-sleeved CGs	(1) Simulated 3 min of double-poling sprints three times. (2) Tested the PO, HR, RER, oxygen uptake, carbon dioxide production, and stroke volume. (3) Tested the blood volume of the triceps brachii with near-IR spectroscopy.	No effect on all parameters.
Wiggen et al., 2016	2016	Thirteen highly trained male cross-country skiers (age: 23 ± 2 years; height: 180 ± 5 cm; body mass: 76.5 ± 4.7 kg; body fat: 9.8 ± 2.0%)	With standard cross-country ski racing suits	(1) At –15 and –6°C, performed Sub1, a self-paced 20-min performance tests, Sub2, and incremental tests to exhaustion. (2) Measured skin and core temperatures, PO, and respiratory variables.	(1) Skin and core temperatures were more reduced with standard racing suits, and average PO was 5% lower in the first 8 min of the performance test at –15°C. (2) From Sub1 to Sub2, the double-poling economy decreased by 3.7% larger at –15°C.
Moon et al., 2016	2016	Ten students (age: 20 ± 0.89 years; height: 172 ± 4.9 cm; body weight: 66.7 ± 10.9 kg; 6 male, 4 female) in a physical exercise-related major	Fabric speed skating suits with three compression levels (0, 9, and 18% downsize)	(1) Surface electromyography test was used to investigate the activation of the RF. (2) The isokinetic test and Wingate test were used to investigate the maximum anaerobic power.	(1) The RF activity was significantly lower at 9% compression than at 0% compression. (2) The mean power and peak torque showed no significant differences in the three compression levels, except the flexion power in 18% compression.
Decker et al., 2016	2016	Nine collegiate alpine ski racers	With DCP or SCP tights	Peak GRF, maximum AP COP, minimum ML COP, COP area, and COP velocity were measured.	(1) During the DCP condition, peak ground reaction force decreased by 9%, the COP was more anterior and medial, and the COP velocity was 14% lower. (2) During the DCP condition, the dynamic balance was improved.
Simons et al., 2016	2016	Nine collegiate alpine ski racers	With DCP or SCP tights	(1) The VL, RF, BF, and GM muscle EMG amplitudes were measured. (2)Average ankle, knee, and hip positions and turn durations were calculated	(1) During the DCP condition, the hip and knee position was more flexed. (2) During the DCP conditions, VL, RF and GM muscle activations were reduced.
**Fatigue recovery (*n* = 1)**					
Govus et al., 2018	2018	Thirty-two Swedish national team skiers (18 males, 14 females)	Commercially available, moderate-pressure, upper- and lower-body CGs	(1) Skiers were randomly assigned to compression garments (*n* = 11), neuromuscular electrical stimulation (*n* = 11), or control group (*n* = 10). (2) CK, urea, CMJ height, and perceived muscle pain were measured before and 8, 20, 44, and 68 h after competition.	Neither CGs nor neuromuscular electrical stimulation promoted physiological or perceptual recovery compared with CON.

**F*_*D*_, drag force; *C*_*D*_, non-dimensional drag coefficient; Re, Reynolds numbers; FT, flow transition; HR, heart rate; VO_2max_, maximal oxygen consumption; VO_2_, oxygen consumption; TTE, running time to exhaustion; LT, lactate threshold; EMG, electromyography; RPE, rating of perceived exertion; CG, compression garment; PO, power output; RER, respiratory exchange ratio; Sub1, a 5-min submaximal test; Sub2, a second 5-min submaximal test; DCP, directional compression; SCP, standard compression; GRF, ground reaction force; AP, anterior-posterior; ML, medial-lateral; COP, center of pressure; VL, vastus lateralis; VM, vastus medial; RF, rectus femoris; BF, biceps femoris; GM, gluteus medius; TA, tibialis anterior; LF, lateral femoral; CK, creatine kinase; CMJ, countermovement jump.*

### Air Drag Reduction

The mechanical energy fluctuations caused by the collision with the external environment during movement leads to changes in the transmission efficiency (Peyre-Tartaruga and Coertjens, 2018). Reduced air drag, in turn, reduces the loss of total mechanical work (Supej et al., 2013). Hence, properly designed CGs with drag reduction feature provide less drag (Supej and Holmberg, 2019). In aerodynamics, the air drag reduction of materials was represented by the drag coefficient (*C*_D_). Air drag (*D*) can be represented as (Oggiano and Sætran, 2010; Chowdhury et al., 2015)

$$D=\frac{1}{2}\,C_{D}\,\rho \,{\mathrm{Av}}^{2},$$

where ρ is the air density, *v* is the air velocity, and *A* is the frontal area of the object. Considering that the human limbs are quasi-cylindrical, the standard cylindrical methodology (Chowdhury et al., 2015) is currently used to quantify the aerodynamic drag on each suit fabric. In 2004, researchers reported a testing of speed skating suit (Brownlie et al., 2004). Using a mannequin model based on anthropometric data from 15 elite sprinters and 16 speed skaters, they tested CGs made from a fitting sample of 39 stretch fabrics. Their results showed that the best-matched fabric reduced air drag by 20.2%. Meanwhile, Oggiano and colleagues conducted a series of studies on the drag reduction performance of CGs by using several parameters, such as position, material, and the stretch of different fabrics (Table 1; Sætran and Oggiano, 2008; Oggiano and Sætran, 2010; Oggiano et al., 2012). Then, a wind tunnel experiment was conducted with a mannequin model based on the average posture of 1500 m speed skating competitors (Sætran and Oggiano, 2008). That study also compared the air drag reduction of fabrics on different body segments, including lower legs, upper legs, trunk, head and arms. On the basis of various parameters (e.g., frontal area and angle), they found that the thighs were the most sensitive area in air drag reduction; mainly, the thighs were perpendicular to the flow with a large frontal area and skin stretching range during movements (Sætran and Oggiano, 2008). Another study tested cross-country ski racing suits and found that rough fabrics could reduce air drag by 10% (Oggiano and Sætran, 2010). Similar to the results of Chowdhury et al. (2015), the C_D_ of the optimal rough fabrics decreased from 0.64 to 0.49 at air velocities of 50–60 km/h on the cylinder model. Considering the surface deformation due to strain after wearing CGs, Oggiano et al. (2012) investigated the air drag reduction of fabrics with different strains. They found that, as the air velocity increased, *C*_D_ was significantly reduced (without values) when reaching the critical velocity (the transition to turbulent); furthermore, with the increase of strain, the critical velocity of rough textile (63% stretched) was closer to smooth textile (23% stretched). That is, a higher strain “smoothens out” the fabric surface and changes the *C*_D_.

The cutting and stitching of suits and other structures have also been shown to be beneficial to drag reduction. For example, the skin suit aligned with the seams corresponding with the air flow direction or were placed in the leeward direction to further reduce air drag (Kuper and Sterken, 2008). Low friction panels were also set under each arm and on the right inner thigh to reduce friction caused by arm swing and pushing off (Kuper and Sterken, 2008). Another skin suit (Braghin et al., 2016) developed by a Dutch company adopted a full-body skating suit design with a “skate strip” (i.e., a smooth rubber material on the head and thighs). Meanwhile, a Japanese company developed new speed skating suits, which reduced turbulence through spiral silicone strips on the thighs and lower arms (Braghin et al., 2016). Researchers tested and modified the different shapes, sizes, and placements of those strips.

Current studies suggest that the air drag reduction mechanism includes the following: (1) theoretically, the remarkable drag reduction property is beneficial to the performance. This can be explained from the energy and efficiency perspective: transmission efficiency is equal to the total mechanical work (*W*_tot_) divided by the positive work produced by the muscle (Peyre-Tartaruga and Coertjens, 2018). Wearing CGs with drag reduction helps reduce *W*_tot_ loss and thus improve transmission efficiency (Supej et al., 2013); (2) a rough surface changes the boundary layer of the cylinder transition from laminar to turbulent in the wake region to reduce pressure drag (Chowdhury et al., 2015), and (3) drag-reducing structures in specific segments can further reduce air drag. However, most studies were laboratory-controlled experiments. The drag reduction effects of CGs in actual competitions remained largely unexplored.

### Metabolism, Muscle Function, and Mechanical Performance

Currently, the main candidate parameters to improve the performance in winter sports disciplines are muscle function, metabolism, and mechanical performance (Hooper et al., 2015; Šambaher et al., 2016; Smale et al., 2018; Hintzy et al., 2019). The primary function of the muscles during locomotion is to produce positive mechanical work. Under the same metabolic energy consumption, the stronger ability to produce positive mechanical work, the higher the muscle efficiency (Peyre-Tartaruga and Coertjens, 2018). The use of CGs seems to provide benefits from these aspects.

Long-distance events in cross-country skiing and speed skating require endurance (Stöggl et al., 2018). Sperlich and colleagues conducted a series of studies (Table 1; Sperlich and Holmberg, 2011; Sperlich et al., 2013, 2014) wherein the physiology of elite cross-country skiers with compression suits was discussed. The subjects wore new (79% polyester, 18% polyurethane, and 3% carbon fabric) and old (80% polyester, 20% elastane) full-body suits. Lower values were found in oxygen uptake, minute ventilation, respiratory exchange rate (RER), heart rate (HR), skin, and core temperature with the new suits (Sperlich and Holmberg, 2011). The authors argued that wearing new suits, with their lighter weight and better moisture-wicking, was more economical for cross-country skiing. The team likewise tested the effect with or without long-sleeve CGs on double-poling sprints (Sperlich et al., 2014). The results showed that compression had no effect on power output, cardiopulmonary parameters, tissue saturation, and blood volume. These findings are consistent with those reported by a previous study on compression shorts for speed skaters (Born et al., 2014).

In terms of muscle function and mechanical performance, researchers have proven that CGs reduce soft tissue vibration (Doan et al., 2003; Hintzy et al., 2019). Alpine skiing can reach top speeds of 140 km/h (Bardal and Reid, 2012) and places high demands on turning and jumping technique for skiers (Jordan et al., 2018). Optimized CGs are required to reduce muscle vibration, especially for sports with high speed and impact. Sperlich et al. (2013) simulated vibration and load in alpine skiing and found that high compression increased knee flexion and decreased muscle vibration and perceived exertion. Furthermore, the EMG of gastrocnemius medialis, rectus femoris, and vastus medialis increased with compression condition. Inconsistent with the aforementioned findings, Moon et al. (2016) reported that leg compression reduced the activation of the rectus femoris in isokinetic tests but had no significant effect on knee extension strength. However, direct comparisons of the findings between these two studies are not appropriate due to the different types of exercise (continuous passive vibrations vs. isokinetic leg extension/flexion) and external pressures adopted (0, 20, and 40 mmHg vs. 0, 9, and 18% compression) Fu et al. (2012, 2015) conducted a series of research on leg compression by testing different muscle contractions (i.e., isometric, isokinetic, and isotonic) and muscle activations for athletes. Their results showed that, while compression shorts did not affect muscle strength acutely, it did reduce the EMG activities and maintained similar power output during repetitive muscle contractions. These results are supported by a recent study from Broatch et al. (2019). Thus, we can speculate that reduced motor unit activation might represent a lower metabolic cost at the molecular level. Using less metabolic cost to maintain the same power output potentially implies higher muscle efficiency (Peyre-Tartaruga and Coertjens, 2018). This proposes another rationale behind the benefits of CGs.

In addition, most researchers held that CGs affected proprioception to achieve optimize performance (Doan et al., 2003; deBritto et al., 2016; Zamporri and Aguinaldo, 2018). Decker et al. (2016) investigated how compression shorts influenced turning direction in alpine skiing by testing the foot pressure characteristics. Their results showed that peak ground reaction force decreased by 9%, the center of pressure (COP) was more anterior, and the COP velocity was 14% lower, all of which demonstrated that leg compression improved dynamic balance. Simons et al. (2016) also analyzed the kinematics and EMG activities of alpine skiing athletes with CGs by performing the experiment in a race simulation. They found that, with compression shorts, the knee and hip flexion increased by 3 and 5%, respectively, and the average vastus lateralis, rectus femoris, and gluteus medius activations decreased by 17, 17, and 26%, respectively. Notably, they first measured the kinematic parameters with inertial measurement units (IMUs) in CGs for winter racing sports. Recently, an image processing system and the IMUs were used for outdoor motion capture (Kruk and Reijne, 2018), specifically for sports with high velocity and huge capture volume. The biomechanical characteristics of winter sports with CGs were further investigated with the help of those methods.

### Fatigue Recovery

Fatigue recovery is crucial for the performance in training and competition for both short- (500 m short-track speed skating) and long-distance (50 km for men and 20 km for women in cross-country skiing) events. Accelerated recovery of energetic substrates can help improve the efficiency of phosphorylative coupling and further enhance muscle efficiency during the movement (Peyre-Tartaruga and Coertjens, 2018). Dealing with fatigue during a match and the recovery after the match are both vital to the performance of athletes. Research have proven that CGs promote fatigue recovery after exercise (Kim et al., 2017; Machado et al., 2018; Nguyen et al., 2019; Pérez et al., 2019).

Govus et al. (2018) tested 32 elite cross-country skiers for blood lactate, creatine kinase, urea, countermovement jumping (CMJ) height, and perceived muscle pain before and after competition. Participants were randomly divided into the CG group (*n* = 11), the neuromuscular electrical stimulation group (*n* = 11), and the control group (*n* = 10). The parameters were tested before and after the competition at 8, 20, 44, and 68 h. Compared with the control group, neither CGs nor neuromuscular electrical stimulation promoted the recovery of blood biomarkers, CMJ height, or perceived muscle pain after competition.

Some limitations in the present study were caused by ecological factors, including dietary habits, intensity difference in experiment and training and competition. Currently, the relevance of the physiology and biomechanics in fatigue and recovery remains unclear. CGs reduce muscle vibration and activation without affecting performance (Fu et al., 2012, 2015; Broatch et al., 2019). Researchers suggested that, for forms of movements that require frequent and repetitive muscle contractions, reduced muscle activation can improve contraction efficiency and decrease energy loss and muscle fatigue. The muscle vibrations and proprioception changes caused by CGs may alter the kinematics of movement, and the characteristics of kinematics are related to the economy and efficiency of motion or the generation of fatigue (Engel et al., 2016). Therefore, there is still a lack of sensitive biomechanical parameters to evaluate CGs for winter sports.

With respect to physiology, CGs have effects on strength promotion, power recovery and reduction of delayed onset of muscle soreness (DOMS) (Bottaro et al., 2011; deGlanville and Hamlin, 2012; Goto and Morishima, 2014; Molly and Shelby, 2018). Furthermore, CGs promote the recovery of exercise-induced muscle damage (Kraemer et al., 2001; Arbabi, 2015), reduced lactate (Duffield et al., 2010; Saulo et al., 2015) and increased tissue saturation (Dermont et al., 2015). Despite the inconsistent results (Bringard et al., 2006; Stickford et al., 2015) regarding the effects of CGs on fatigue performance and economy of motion, this may be caused by the heterogeneity of the test procedures, e.g., the difference of types and levels of compression (Pérez et al., 2019).

Furthermore, different sports have different techniques and tactical characteristics that indicate different demands on CGs for fatigue recovery. Thus, the use of CGs for specific winter sports is worthy of further exploration and should not be limited to speed skating, cross-country skiing, and alpine skiing. Moreover, systematic reviews of the role of compression in major physiological aspects in winter racing sports, e.g., fatigue recovery and muscle function enhancement, are warranted.

## Conclusion

Winter sports have increasingly relied on professional equipment, such as CGs. The main points held by this narrative review include the following. (1) Currently, CG studies in winter racing sports have mainly focused on drag reduction, metabolism, muscle function, strength performance, and fatigue recovery. (2) The results of most studies conducted in wind tunnels showed that, for cylindrical structures similar to the human body, clothing with rough surfaces can reduce air drag. Notably, the drag reduction effect of CGs in real competition have yet to be fully explored. (3) Compression can reduce muscle vibrations at high impact and help athletes control the COP movement during competition, a function that is important for alpine skiing. However, it is noteworthy that, for the effects of CGs, the number of studies on winter racing sports is not as many as those on general sports.

Future studies could focus on the following subtopics: (1) investigating the effects of clothing microstructure on drag reduction and their stretching in different parts of the body, specifically on which area is most sensitive to reducing air drag; (2) ensuring that the temperature, humidity, and speed during experimental research are consistent with those during the competition; and (3) providing a full discussion on energy metabolism, fatigue, and recovery affected by CGs. These effects cannot be ignored, because long-distance events are carried out in harsh environments and changing tracks.

## Author’s Note

This is a narrative review of past studies that are relevant to the topic of interest.

## Author Contributions

WF contributed to the conceptualization, project administration, and funding acquisition. CY and YX contributed to the literature retrieval and the preparation writing of the original draft preparation. CY, YX, YY, and SX contributed to the literature screening. YX contributed to the data curation. WF, CY, YX, YY, and SX contributed to the review and editing. All authors contributed to the article and approved the submitted version.

## Conflict of Interest

The authors declare that the research was conducted in the absence of any commercial or financial relationships that could be construed as a potential conflict of interest.
